# An analysis on the effect of the three-incision combined approach for complex fracture of tibial plateau involving the posterolateral tibial plateau

**DOI:** 10.1186/s13018-020-1572-4

**Published:** 2020-02-11

**Authors:** Guqi Hong, Xiaowen Huang, Tianrun Lv, Xiang Li

**Affiliations:** Department of Orthopedics, First Affiliated Hospital of Nanjing Medical University (Jiangsu Province Hospital), Nanjing, 210029 People’s Republic of China

**Keywords:** Tibial fracture, Knee joint, Internal fracture fixation, Combined incision

## Abstract

**Background:**

The clinical effect of the three-incision combined approach for complex fracture of tibial plateau involving the posterior tibial plateau was discussed.

**Methods:**

A retrospective analysis was performed for 13 cases receiving surgery for complex fracture of tibial plateau from July 2015 to June 2019. They received surgery via the three-incision combined approach, and regular postoperative reexamination was performed at the outpatient clinic. During the last follow-up, Hospital for Special Surgery (HSS) Knee Scoring System was used to assess the knee joint function; the Lysholm score was used to assess the knee mobility. The anterior, posterior, and rotational stabilities of the knee joint were assessed by the Lachman test and pivot-shift test.

**Results:**

There was no nonunion and delayed union, implant loosening and fracture, or refracture, and neither were there neurological symptoms or restricted mobility in daily life. During the follow-up, none of the cases were found with restriction of knee mobility caused by internal fixation or apparent pain. The HSS score during the last follow-up was 86–100 (average, 90.2 ± 6.8), and the excellent and good rate was 100%; the Lysholm score was 86–100 (average, 95.7 ± 2.6). All cases were negative for the Lachman test and pivot-shift test. The knee flexion mobility was 100~140° (average, 127.2° ± 11.4°). Postoperative X-ray indicated anatomical reduction of bone fractures in all cases. Loss of reduction or loosening and fracture of internal fixation was not observed by postoperative regular reexaminations. The posterior tibial slope at 6 months after surgery was 6~16° (average, 10.66 ± 2.58°), the varus angle was 84~89° (average, 86.52 ± 1.46°), the Rasmussen radiological score was 12~18 (average, 16.12 ± 1.35), and the excellent and good rate was 100%.

**Conclusion:**

The three-incision combined approach proved safe and reliable for complex fracture of tibial plateau involving the posterior tibial plateau and is worthy of further popularization.

## Background

Tibial plateau fracture is a typical intra-articular fracture, which accounts for 1.9–4% of all bone fractures [1]. Due to anatomical complexity, poor treatment will greatly influence knee joint stability and function, and a high requirement is hence placed on reduction and fixation. Complex tibial plateau fracture is usually a high-energy injury which is difficult to treat and associated with a variety of postoperative complications. The surgical treatment for this condition is still a controversial topic [2, 3] and remains one of the major challenges among the traumatic fractures.

Classification of bone fractures is an important guiding principle for the treatment of complex tibial plateau fracture. The incidence of complex tibial plateau fracture involving the posterior tibial plateau was once believed to be very low, and this condition did not draw enough attention. Neither the conventional Schatzker classification system nor AO classification provided a systematic description of this type of fracture. But along with the deepened understanding of tibial plateau fracture as well as the progress in medical technology such as the extensive application of 2D and 3D reconstruction of CT images, many patients are found with tibial plateau fracture involving the posterior tibial plateau. However, the reduction and fixation of posterior fracture blocks are much more difficult. If not properly treated, the posterior fracture blocks can cause a significant increase in local stress, severe secondary wear, and degeneration of the articular cartilage, which greatly influences the knee joint function and daily life of the patients. Therefore, the conventional Schatzker classification and AO classification can no longer meet clinical needs. Long et al. proposed the three-column classification theory for tibial plateau fracture on this basis [4, 5], which described the tibial plateau fracture, especially the fracture of the posterior tibial plateau, so as to better guide the clinical treatment. For posterior column tibial plateau fracture, the conventional surgical approaches include Carlson’s approach and inverted L-shaped approach. But when the fracture involves all three columns, the limitations of the above conventional surgeries become pronounced. For example, it is difficult to adjust the body position during surgery, the surgical field is insufficiently exposed, and important blood vessels and nerves are easily damaged [6, 7]. For a long time, many scholars have attempted to modify the surgical approaches for this type of bone fractures, for example, modifying the anterolateral approach [8] and fibular osteotomy approach [9]. These approaches are mainly used to treat fractures involving the lateral and posterolateral tibial plateau. But for fractures simultaneously involving the medial tibial plateau or having larger posterior fracture blocks that involve the posteromedial side, these approaches can hardly achieve effective reduction and fixation while reducing the invasiveness.

Based on the classical Carlson’s approach and inverted L-shaped approach, modifications were done to the conventional anteroposterior and anteromedial approaches. We innovatively proposed a three-incision combined approach to treat this type of tibial plateau fracture, and a retrospective analysis was conducted for the following purposes: (1) to determine the indications for the three-incision combined approach, (2) to assess the feasibility and short-term efficacy of the three-incision combined approach for the complex tibial plateau fracture involving the posterior column, and (3) to summarize the advantages and defects of the three-incision combined approach.

## Materials and methods

### Inclusion and exclusion criteria

The inclusion criteria were as follows: (1) adults (aged over 18 years old) with fresh tibial plateau fracture; (2) confirmed as type V tibial plateau fracture involving the posterior tibial plateau according to the Schatzker classification system by preoperative X-ray and CT; (3) no apparent vascular and nerve damage before surgery; (4) no preoperative incision infection or destructive injury, with soft tissue conditions allowing for internal fixation; (5) no apparent preoperative surgical contraindications and tolerable to surgery; and (6) postoperative follow-up ≥ 6 months.

The exclusion criteria were as follows: (1) open tibial plateau fracture, (2) multiple bone fractures in the lower limbs interfering with subsequent rehabilitation exercise, (3) unable to cooperate with rehabilitation therapy after surgery, and (4) having a history of knee osteoarthritis or rheumatoid arthritis, which might interfere with postoperative functional evaluation of knee joint.

### Baseline data

Thirteen cases with complex tibial plateau fracture and receiving surgery from July 2015 to June 2019 were recruited. There were eight males and five females, who were aged 24–68 years old, with an average of 40.7 ± 14.2 years. The causes of fractures were traffic-related injuries, and all fractures were closed. Preoperative examinations did not reveal vascular and nerve injuries. There were no combined injuries at other sites.

All of them received preoperative X-ray and CT plain scan+3D reconstruction. Two cases had suspected cruciate ligament injury, which was excluded after MRI. According to Schatzker classification, all of them were of type V. For AO/OTA classification, all of them were of type 41B-3.1. According to the three-column classification theory by Carlson et al. [6], all fractures involved the lateral, medial, and posterior columns.

The present study was approved by the institutional ethics committee, and all cases signed the informed consent.

### Preoperative preparation

The soft tissue conditions were carefully evaluated before surgery. Mannitol or sodium aescinate was given as routine detumescence therapy. The patients were told to elevate the affected limb and exercise the toes and ankle joints. Eight cases received surgery when the swelling basically subsided at about 7 days after injury; five cases with severe soft tissue swelling received surgery at 10 days after injury. For the latter, there might be a risk of deep vein thrombosis in the lower limbs; for those aged above 60 years old, the risk of thrombosis was higher and low-molecular-weight heparin should be given before surgery. If thrombosis was suspected, bedside lower limb venous ultrasonography should be immediately performed for confirmation. Two cases were found with deep venous thrombosis in the lower limbs before surgery and received inferior vena cava filter implantation, followed by surgery for tibial plateau fracture.

### Surgical procedures (Fig. 1)

The patient took a floating position and received general anesthesia or epidural anesthesia. Tourniquet was applied to the thigh root.

**Fig. 1:**
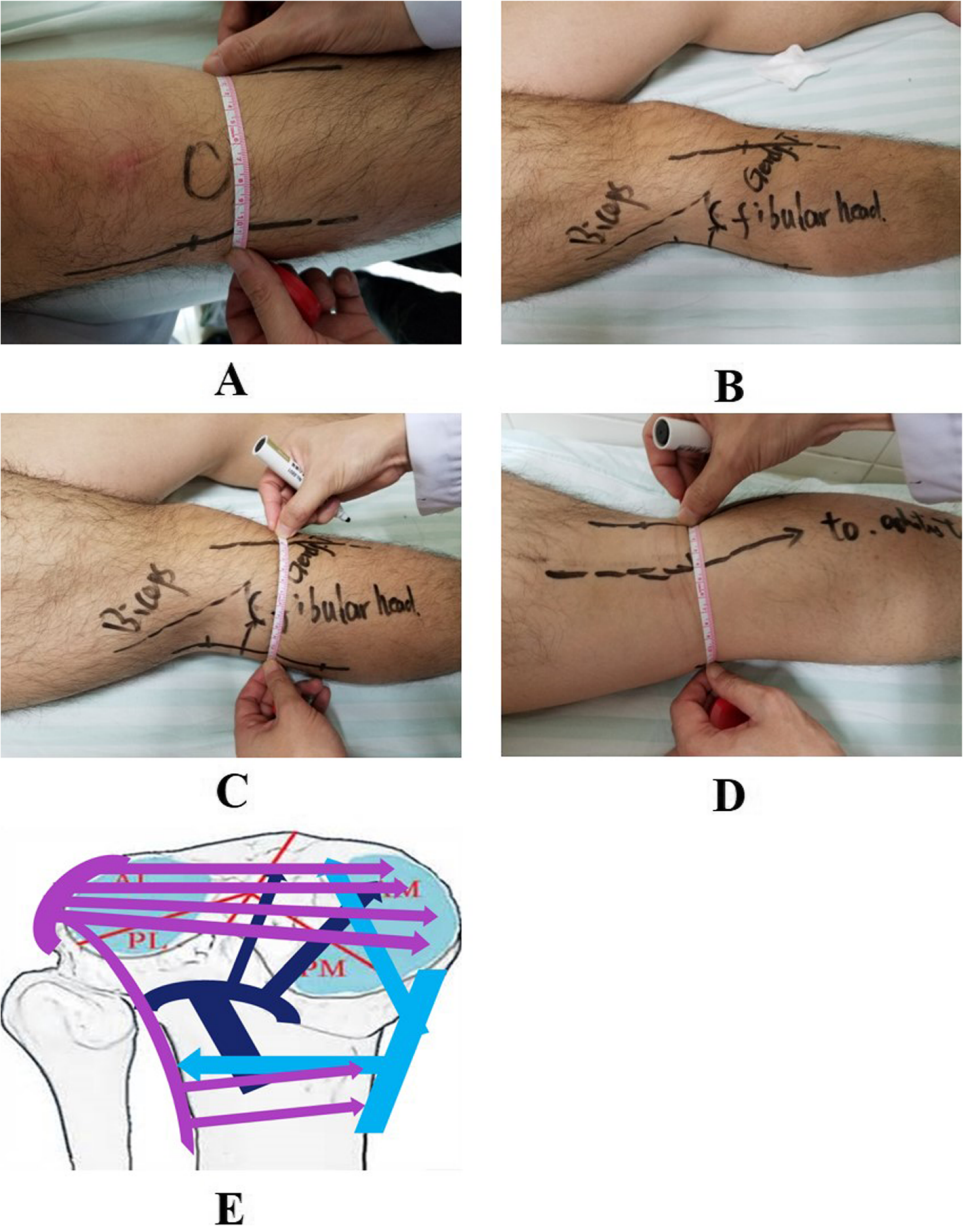
Surgical procedures. **a**–**d** Design of the three incisions. **e** Reduction of fracture and internal fixation

The patient took a semiprone position during surgery. Posterolateral approach was adopted. A longitudinal incision of about 10 cm was made along the midpoint between the posterolateral margin of fibular head and the mid line of the popliteal space. The proximal end extended to the posterolateral margin of the biceps femoris muscle. The lateral head of gastrocnemius was retracted medially, and the vascular branches on the surface of the soleus was carefully treated and peeled off. Attention was given to protect the popliteus tendon, and the lateral wall of the tibial plateau was exposed. It is not necessary to isolate and expose the common peroneal nerve during surgery. Under the anterolateral approach, an incision with a diameter of 6–8 cm was made from the lateral side of the lower pole of the patella towards the distal end through the space between the Gerdy’s tubercle and tibial tubercle. The anterolateral tibial plateau was cut open and isolated layer by layer. The articular surface was exposed after opening the articular capsule, and the lateral meniscus was examined and protected. By reference to the anatomical landmark in the posterolateral wall and under the direct view of the normal articular surface on the anterolateral side, the lateral column, fracture block of the posterior column, and collapsed articular surface were reduced. If there was comminuted fracture on the lateral side, a small bone window would be made within the posterolateral incision at 1–2 cm from the articular surface. The collapsed articular surface was jacked up with an osteotome. Under the direct view, the height of the articular surface was completely restored. The bone defect was filled with allogeneic cancellous bone strip or autologous iliac bone with compaction. The posterior fracture blocks were first fixed using T-shaped steel plates for distal radius bone or one-third tubular plate in the posterolateral incision; then, fixation was performed using the L-shaped anatomical locking plate in the anterolateral incision (3.5 mm, raft). Under the direct view, it was confirmed that no screws entered the articular cavity. The position of the plate was determined as satisfactory by X-ray, and the screw length was appropriate.

For posteromedial tibial plateau fracture, the medial fracture blocks were exposed via the posterolateral incision in 12 cases. The posteromedial tibial plateau fracture was reduced based on the anatomical relationship of posteromedial cortical bone. Then, the patient was changed to a supine position. The classical anteromedial approach to the knee joint was adopted [10]. An incision of 6–8 cm was made. After exposing the fracture blocks in the medial column of the tibial plateau layer by layer, a long straight locking compression plate or one-third tubular plate was used to reinforce the fixation. For 1 obese patient with diabetes, due to soft tissue shielding behind the knee joint, it was difficult to achieve precise reduction through the posterolateral incision. Instead, the posterior fracture was directly reduced and fixed via the medial approach.

The reduction of fracture was assessed by X-ray again. All implant positions and screw lengths were satisfactory. Then, ADT test, Lachman test, and varus-valgus rotation were performed to ensure good stability of the knee joint, tight internal fixation, and no entrapment of the joint. The incision was washed with a large amount of normal saline. One drainage tube was placed via the medial incision, and no drainage tube for the anterolateral and posterolateral incisions.

### Postoperative exercise and rehabilitation training

The patients were told to elevate the affected limb immediately after surgery. Quadriceps isometric contraction exercise and ankle knee extension exercise began at 1 day after surgery, which was conductive to detumescence and to preventing deep venous thrombosis in the lower limbs. After the swelling of the knee joints subsided at about 1 week after surgery, the patients began to do passive keen flexion exercise through continuous passive motion (CPM). The incisions basically healed at about 2 weeks after surgery, and the patients began to take active knee flexion exercise. The patients achieved knee flexion of 90° and above within 1 month of progressive training. Reexaminations were performed by X-ray every 4–6 weeks after surgery. Based on the reexamination results at 2–3 months after surgery, the patients began to do weight-bearing exercise if the bone fracture healed well until full weight-bearing.

### Follow-up and efficacy evaluation indicators

The patients received regular reexaminations at the outpatient clinic at 1, 3, and 6 months after surgery, respectively. The bone fracture reduction and fixation conditions of the knee joint were assessed by X-ray in the anteroposterior and lateral views, and the knee joint functions were determined.

They were assessed by Rasmussen radiological scores based on X-ray [11], and the changes in posterior tibial slope and varus angle were measured. During the last follow-up, Hospital for Special Surgery (HSS) Knee Scoring System [12] was used to assess the knee joint function; the Lysholm score [13] was used to assess the knee mobility. The anterior, posterior, and rotational stabilities of the knee joint were assessed by the Lachman test and pivot-shift test.

The Rasmussen criteria for radiological assessment were as follows: Assessments were performed from the perspectives of plateau collapse and widening and genu varum and genu valgum. The full score was 18, indicating excellent; the score of 12–17 indicated good, 6–11 moderately good, and < 6 poor.

HSS scoring criteria involved the following dimensions: pain (30), function (22), mobility (18), muscle tone (10), flexion deformity (10), and stability (10). The total score was 100, and the score ≥ 85 was defined as excellent, 70–84 scores good, 60–69 fair, and < 59 poor.

The Lysholm criteria were as follows: whether there was walking instability, whether there was pain when walking, whether there was swelling when walking, whether there was claudication, and whether there was the need for support during weight bearing, stair climbing, and squatting. The full score was 100, and the higher the score, the better the knee joint function was.

### Statistical analysis

SPSS18.0 software (SPSS, USA) was used for statistical analysis. Measurement data were expressed as mean ± standard deviation. Immediately after surgery and at 6 months after surgery, the varus angle of tibial plateau of the affected limb, posterior tibial slope, and Rasmussen radiological score were compared by the paired-samples *t* test. The significance level *α* was set as 0.05 (two-sided).

## Results

### General results

The time from injury to surgery was 6–14 days in 13 cases, with an average of 7.6 ± 1.8 days. The surgical time was 1.9~3.2 h, with an average of 2.4 ± 0.6 h; the intraoperative blood loss was 180–400 ml, with an average of 310 ± 74 ml. Among them, 12 cases achieved grade I/A incision healing within 10.8 ± 2.4 days on average; the remaining 1 obese case (BMI > 30) with diabetes suffered from fat liquefaction of incision, the incision healing was of grade I/B, and it took 21 days for the incision to heal.

### Follow-up results

The postoperative follow-up duration was 5–42 months in all patients, with an average of 21.4 ± 2.8 months. The time to full weight-bearing after surgery was 2–3 months, with an average of 2.4 ± 0.6 months; the time to bone fracture healing was 10–18 weeks, with an average of 14.2 ± 2.5 weeks. There was no nonunion and delayed union, implant loosening and fracture, or refracture after surgery. During the follow-up period, none of the cases was found with restriction of knee mobility caused by internal fixation or apparent pain. Two cases had the internal fixation removed at 1.5 and 2 years after surgery, respectively.

### Evaluation of clinical knee joint functions

Reexamination at the outpatient clinic at 6 months after surgery indicated no restricted mobility in daily life in none of the patients. All of the patients were negative for the Lachman test and pivot-shift test. The knee flexion mobility was 100~140°, with an average of 127.2° ± 11.4°. The HSS score during the last follow-up was 86–100, with an average of 90.2 ± 6.8, and the excellent and good rate was 100%; the Lysholm score was 86–100, with an average of 95.7 ± 2.6.

### Radiological assessment

**Fig. 2:**
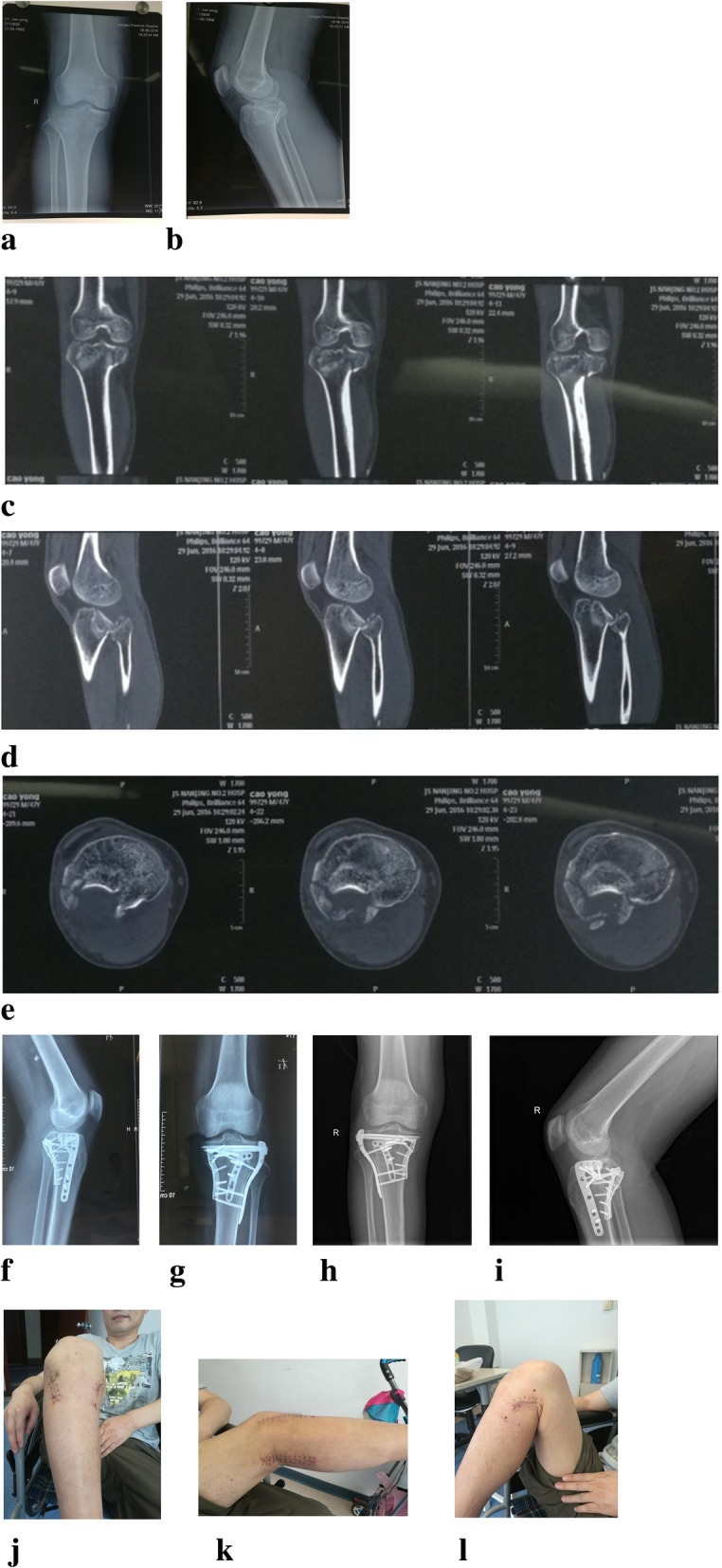
**a**–**c** A typical case of the three-incision combined approach for complex fracture of tibial plateau involving the posterolateral tibial plateau. A male 41 case suffered traffic accidents, and preoperative X-ray indicated tibial plateau fracture, which was of type V according to Schatzker classification. Further CT examination indicated the fracture affected all the three columns, and three-incision combined approach for fracture reduction and fixation was performed. Follow-up results at 1 year after surgery showed good recovery of the patient, with good flexion and extension of the knee joint. **a**-**b**: pre-op X-ray; **c**-**e**: pre-op CT; **f**-**g**: post-op X-ray immediate; **h**-**i**: post-op X-ray after one year follow-up; **j**-**l**: Functional posture image one year follow-up after operation

Postoperative X-ray indicated that all patients achieved anatomical reduction of the fractured sites. All of them were examined regularly by X-ray in the anteroposterior and lateral views at the outpatient clinic. Among the 13 patients, the posterior tibial slope immediately after surgery was 5~14°, with an average of 10.13° ± 2.69°; the varus angle was 85~88°, with an average of 87.00° ± 1.21°; the Rasmussen radiological score was 14~18, with an average of 16.92 ± 1.68, and the excellent and good rate was 100%. The posterior tibial slope at 6 months after surgery was 6~16°, with an average of 10.66 ± 2.58°; the varus angle was 84~89°, with an average of 86.52 ± 1.46°; the Rasmussen radiological score was 12~18, with an average of 16.12 ± 1.35, and the excellent and good rate was 100%. There were no significant differences in the reexamination results immediately after surgery and at 6 months after surgery. For more details, see Table 1. The typical case is illustrated in Fig. 2.

**Table 1:** Radiological assessment of the index

	Posterior tibial slope	Varus angle	Rasmussen radiological scores
Immediately after surgery	10.13 ± 2.69	87.00 ± 1.21	16.92 ± 1.68
6 months after surgery	10.66 ± 2.58	86.52 ± 1.46	16.12 ± 1.35
Difference	0.53 ± 0.79	0.43 ± 0.53	0.29 ± 2.76
*t*	1.441	2.121	1.000
*P*	0.20	0.078	0.356

### Complications

The above-mentioned 1 obese patient with diabetes suffered from fat liquefaction of incision. No abnormal elevations of the white blood cell count, erythrocyte sedimentation rate, and C-reactive protein level were observed. Debridement was performed once at 7 days after internal fixation, followed by dressing change and infrared radiation of the incisions. The incisions gradually healed, and incision infection did not occur. None of the 13 patients had incision skin margin necrosis or nerve injury symptoms in the lower limbs. Two patients had swelling and pain in the affected limb before surgery, and they were positive for the Homan’s sign (+). These 2 cases were found with deep vein thrombosis in the lower limbs by venous ultrasonography before surgery and received inferior vena cava filter implantation, followed by surgery for tibial plateau fracture. Complications such as hypostatic pneumonia or bedsores did not occur. During the follow-up, there was no loosening or fracture of the screws, loss of reduction, nonunion, or delayed union.

## Discussion

### Injury mechanism and classification

Along with social development and changes of people’s daily life habits, an increasing number of patients have bone fractures involving the posterior tibial plateau. It has been reported that the incidence of bone fractures involving the posterior tibial plateau is 14.8~44.2% [14, 15]. The conventional Schatzker classification system does not describe the bone fractures involving the posterior tibial plateau, while the AO/OTA classification system is too complex [1]. Luo et al. [4] proposed the three-column classification for tibial plateau fracture. On this basis, Chen et al. [16] performed further classification of the fractures involving the posterior tibial plateau by combining with CT reconstruction of the 3D images. Seven patients in the present study belonged to type V bone fractures involving the two condyles according to the Schatzker classification system. They were of type V according to the classification system proposed by Chen et al., that is, cleavage fracture and collapse of the lateral condyle involving the posterior tibial plateau and combined with cleavage fracture of the medial condyle. As to the injury mechanism, this type of fracture is mainly caused by the impact to the tibial plateau and femoral condyles under vertical or varus and valgus stresses with the knee joint in flexion or “semiflexion” [17].

### Selection of implants for the studied approach and fixation

For complex tibial plateau fractures involving both the medial and lateral condyles, the reduction and fixation of medial tibial plateau fracture are usually performed first. This is because the fracture blocks in the medial tibial plateau are more intact and therefore easier to be reduced and fixed. Then, the reduction and fixation of the lateral tibial plateau is performed by reference to the medial tibial plateau. The fractures in our patients affected all of the three columns, namely, medial, lateral, and posterior, which needed to be reduced and fixed. To facilitate intraoperative shift in body position, the patients initially took the floating position with change from semi-prone to supine. Under this body position, it would be more convenient to perform the reduction and fixation of the anterolateral and posterolateral tibial plateau fractures first. Moreover, the two incisions, one anterior and the other posterior, allowed for a sufficient exposure of lateral tibial plateau fractures as well as easier performance of reduction and fixation. As the fixation of fractures involving the lateral condyle and posterior tibial plateau, we believe that the fracture blocks in the posterior tibial plateau should be first fixed and then those in the lateral condyles to prevent loss of reduction. When fixing the fracture blocks in the posterior tibial plateau using the steel plate, supporting the fracture blocks is just enough. For simple posterior tibial plateau fracture, one-third tubular plate is sufficient for the fixation. But for more complex fractures, we believe that the T-shaped steel plate for distal radius bone is a better choice. This plate has a smaller volume and causes less irritation to soft tissues. When using the above-mentioned approach, a large manipulation space is allowed in the posterior, thus reducing the adverse impact from the shielding of fibular head. Neither is a need for fibular osteotomy, thus reducing the risk of side injuries and postoperative posterolateral instability of the knee. When doing fixation using the T-shaped steel plates for distal radius bone, the medial fracture blocks are fixed with screws after the T-shaped support, while the lateral fracture blocks are only shielded without the use of screws. The L-shaped anatomical locking plate for the lateral condyle via the anteroposterior incision offers robust fixation, while raft fixation is performed for the proximal end. For medial condyle fixation, a straight locking compression plate was used for the medical incision of the tibial plateau to reinforce the fixation. During reduction of the lateral tibial plateau fracture via the anterolateral incision, the cortex between the fracture blocks was used as the anatomical landmark. Anatomical reduction could be performed under direct view, and the effect of reduction of the lateral tibial plateau was assessed by intraoperative X-ray. The L-shaped anatomical locking plate was chosen for the lateral tibial plateau after reduction to offer robust fixation, while raft fixation was performed for the proximal end. For medial condyle fracture, reduction was performed via the medial incision in a supine position. The reduction effect was assessed by X-ray by reference to the lateral tibial plateau. After confirming that the reduction was successful, a straight locking compression plate was used to strengthen the fixation. For fractures with insignificant medial condyle displacement, the one-third tubular plate can be also chosen.

### Comparison with other approaches

When it comes to the choice of surgical approach for fractures involving the posterior tibial plateau, the conventional approaches include the inverted L-shaped approach, Carlson’s approach, and anterolateral approaches. But these approaches have many defects in clinical applications. The inverted L-shaped approach and Carlson’s approach are usually performed in a prone position. When fractures involve both the anterior and posterior aspects of tibial plateau, it is difficult to perform reduction and fixation of fracture in the anterior tibial plateau in the prone position alone. Thus, it is necessary to adjust the body position, which increases the complexity of surgery. Moreover, Carlson’s approach involves complex anatomical structure and easily damages the blood vessels and nerves. It is difficult to visualize the articular surface under naked eyes. Since there are no anatomical landmarks, it is difficult to perform reduction for the posterior collapse fracture [4]. In some studies, the articular surface is exposed by severing the popliteus muscle, though the exposure is not sufficient. Moreover, improper manipulation can easily cause disturbance to knee varus and extorsion and back and forth movement of tibia [18]. The inverted L-shaped approach is highly invasive for the soft tissues. Due to the shielding from the medial head of the gastrocnemius, it is very difficult to perform exposure, reduction, and fixation of the posterolateral tibial plateau fracture. Since the articular surface cannot be visualized under the naked eyes, this approach has some defects when treating the collapse of posterolateral tibial plateau. Tao et al. [19] reported that among 11 patients with tibial plateau fracture treated by the posterior inverted L-shaped approach, 5 patients showed mild knee inflexion and contracture. When exposing the posterior fracture blocks via the anterolateral approach, it is necessary to perform fibular osteotomy during surgery due to shielding from the fibula. This operation is likely to damage the common fibular nerve. Moreover, as more soft tissues are peeled subcutaneously during the exposure, the invasiveness is greater, which increases the risks of postoperative incision infection and necrosis [9]. This approach can hardly treat the posterior to medial fractures, and the scope of indications is narrow [20].

We adopted the three-incision combined approach after analyzing the respective advantages and disadvantages of the existing approaches. This approach was the modification and combination of the conventional anterolateral approach, Carlson’s posterolateral approach, and medial approach. By analyzing 13 cases included, it was found that this approach had seven advantages: ① The patients took a floating position. First, it was the semiprone position and then the prone position. When changing the body position, all that was needed was to move back and forth slightly, which was a simple operation and convenient to expose the incision. ② Under one body position, the anterolateral and posterior aspects of the tibial plateau could be exposed simultaneously, which fully exposed and reduced nearly all types of tibial plateau fractures except for medial condylar fracture. One additional medial incision for treating medial condylar fracture was sufficient to treat all fractures. ③ All three incisions under this approach were straight incisions, between which the distances were nearly equal. This would greatly reduce the skin tension of incision, and it was not necessary to excessively peel off the subcutaneous soft tissues. By doing this, the damage to the blood supply to the fractured end and postoperative incision infection, necrosis, and long-term scar contracture could be greatly reduced. As a result, the actual invasiveness of this approach was small. Even with three incisions, the surgical time and intraoperative blood loss were not increased, and the incisions healed well. ④ The posterolateral incision in this combined approach was closer to the medial aspect than conventional incisions, while the anterolateral incision was closer to the anterior aspect than conventional incisions. Thus, the common fibular nerve was avoided. There was enough space to expose the posterolateral aspect of the knee joint under naked eyes, which was conducive to avoiding damage to important structures such as vascular branches and posterior cruciate ligament and hence reducing iatrogenic injury. ⑤ All manipulations of fracture reduction and fixation under this approach could be performed under naked eyes, which was easy, convenient, causing fewer intraoperative side injuries, and reducing the number of intraoperative X-ray scans. ⑥ Thin steel plates were usually used for the posterior and medial aspects, thus causing lower irritation to the soft tissues. ⑦ Unlike the conventional procedure of first reducing and fixing the medial tibial plateau fracture, we first reduced the posterior and lateral tibial plateau fractures via this approach, thus achieving multi-plane stereotactic fixation. Then, the medial condylar fracture blocks of the tibial plateau were fixed according to the anatomical landmarks. Complete anatomical reduction could be achieved for all fractures, and none of our cases lost the reduction through postoperative exercises.

### More about bone grafting

For collapse fracture of the articular surface, gaps associated with bone defects will still exist after reduction. In the present study, all seven cases with such condition were treated by bone grafting. Allogeneic cancellous bone strip was used in five cases, and autogenous iliac bone implantation was performed in two cases. Postoperative follow-up and reexamination indicated good fracture healing. The primary purpose of intraoperative bone grafting is to provide a good support for the articular surface and to avoid recollapse of the articular surface after surgery. During screw fixation, the screw contact area is increased to enhance the stability of fixation. This method can also eliminate the dead space and prevent infection.

### About the removal of implants

Our three-incision combined approach is still invasive for complex tibial plateau fracture. We believe that it is not necessary to remove the internal fixation plates and screws so long as the patients have no significant restriction of mobility or pain caused by the implants. Two cases had the internal fixation removed at 1.5 and 2 years after surgery, respectively, due to their strong request. The internal fixation was not removed for the remaining cases. When removing the implants, all three plates and screws can be exposed and removed by opening the original anterolateral and medial incisions.

### Limitations and defects of the present study

Only 13 cases were recruited in the present study, and the sample size was small. The follow-up was short, and long-term follow-up is needed to summarize clinical experience. Furthermore, the simple medial incision involved in our approach can achieve effective reduction and fixation of the cleavage fracture of the medial condyle of tibial plateau. But for fractures involving the collapse of the medial tibial plateau, the reduction will be more difficult. So far, we have no experience in the treatment of this type of patients, and more work should be done in this respect.

## Conclusion

Our study showed that the three-incision combined approach was safe and reliable for complex fracture of tibial plateau involving the posterolateral tibial plateau. The intraoperative manipulations were simple, with sufficient space and few side injuries. The postoperative complications were few, and the patients could achieve fast postoperative recovery. This approach is worthy of further clinical applications.

## Data Availability

All data analyzed during this study are included in this published article.

## Abbreviations

AO: Arbeitsgemeinschaftfür Osteosynthesefragen
BMI: Body mass index
CPM: Continuous passive motion
CT: Computed tomography
HSS: Hospital for Special Surgery
OTA: The Orthopaedic Trauma Association
